# Developing Questions to Assess and Measure Patients’ Perceived Survival Benefit from Adjuvant Endocrine Therapy in Breast Cancer: A Mixed Methods Pilot Study

**DOI:** 10.21203/rs.3.rs-3260720/v1

**Published:** 2023-08-18

**Authors:** Bernard Tawfik, Kendal Jacobson, Ursa Brown-Glaberman, Mikaela Kosich, M. Lee Horn, Jacklyn Nemunaitis, Zoneddy Dayao, V. Shane Pankratz, Andrew L. Sussman, Dolores Guest

**Affiliations:** University of New Mexico Comprehensive Cancer Center; University of New Mexico Comprehensive Cancer Center; University of New Mexico Comprehensive Cancer Center; University of New Mexico Comprehensive Cancer Center; University of New Mexico College of Education and human Sciences; University of New Mexico Comprehensive Cancer Center; University of New Mexico Comprehensive Cancer Center; University of New Mexico Comprehensive Cancer Center; University of New Mexico Comprehensive Cancer Center; University of New Mexico Comprehensive Cancer Center

**Keywords:** Breast Cancer, Adjuvant Endocrine Therapy, Patient Understanding, Overall Survival Benefit, PREDICT Model

## Abstract

**Purpose:**

This mixed methods study developed multiple question types to understand and measure women’s perceived benefit from adjuvant endocrine therapy. We hypothesis that patients do not understand this benefit and sought to develop the questions needed to test this hypothesis and obtain initial patient estimates.

**Methods:**

From 8/2022 to 3/2023, qualitative interviews focused on assessing and modifying 9 initial varied question types asking about the overall survival (OS) benefit from adjuvant endocrine therapy. Subsequent focus groups modified and selected the optimal questions. Patients’ self-assessment of their OS benefit was compared to their individualized PREDICT model results.

**Results:**

Fifty-three patients completed the survey; 42% Hispanic, 30% rural, and 47% with income <$39,999 per year. Patients reported adequate health care literacy (61.5%) and average confidence about treatment and medication decisions 49.4 (95% CI 24.4–59.5). From the original 9 questions, 3 modified questions were ultimately found to capture patients’ perception of this OS benefit, focusing on graphical and prose styles. Patients estimated an OS benefit of 42% compared to 4.4% calculated from the PREDICT model (p < 0.001).

**Conclusion:**

In this group with considerable representation from ethnic minority, rural and low-income patients, qualitative data showed that more than one modality of question type was needed to clearly capture patients’ understanding of treatment benefit. Women with breast cancer significantly overestimated their 10-year OS benefit from adjuvant endocrine therapy compared to the PREDICT model.

## Introduction

Breast cancer is the most common cancer in the United States and modern therapies are very effective with cure rate in excess of 80% [1, 2]. Adjuvant endocrine therapy is a mainstay of treatment for the 70% of breast cancer patients whose breast cancer expresses Estrogen Receptors (ER) and/or Progesterone Receptors (PR) [3]. Adjuvant endocrine therapy has been shown to be effective at reducing breast cancer recurrence and improving overall survival (OS) [4]. However, compliance has long been an issue, with only 60% of patients completing the recommended 5 year course of adjuvant endocrine therapy even though higher compliance rates have been shown to improve outcomes [5, 6]. Medication compliance is also worse among Hispanic patients and those living in rural areas [7, 8]. Poor compliance leads to increased recurrence and mortality, especially in those with locally advanced disease [9, 10].

Conversely, over 90% of women with early stage breast cancer suffer from significant side effects related to adjuvant endocrine therapy and a subset of these women derive only a small benefit [11, 12]. For example, a 65-year-old postmenopausal woman with an estrogen receptor positive, Her2 negative, 1.5 cm breast cancer with no nodal involvement, the 10 year absolute OS benefit from adjuvant endocrine therapy is 0.9% per the PREDICT model [13]. That same example with a 5.5 cm tumor and 4 lymph nodes involved shows a benefit of 6.2%. Women with breast cancer may not understand the relatively small benefit of their therapy on survival, as it is well known that patients do not understand many aspects of their medications in general including the actual benefits [14–16]. It has been previously shown with other breast cancer treatment decisions that patients trust their physicians to provide the best treatment recommendations, so this may also impact patients’ choices to not discontinue their medication despite significant side effects [17]. In addition, perceptions of risk have also been shown to be related to behaviors including compliance, thus understanding how women view their risk and benefit from adjuvant endocrine therapy is important for decision making [18]. Additionally, women from ethnic minority populations and those of lower socioeconomic status often have lower health care literacy in general and thus understanding the benefit from adjuvant endocrine therapy is especially important for these women as they have a higher risk of an inaccurate perception [7, 19].

PREDICT is a validated online tool that estimates absolute OS benefit from adjuvant endocrine therapy and is widely used [20]. Numerous validated questionnaires exist to assess patient understanding and side effect burden of medication in general but not to assess patient understanding of the perceived benefit from adjuvant endocrine therapy specifically [21]. Data visualization is recognized as an important avenue to assess and disseminate medical information with patient and providers and research for the optimal patient visualization strategy is ongoing [22, 23].

We hypothesized that women with early stage breast cancer do not accurately understand the expected OS benefit of their adjuvant endocrine therapy, particularly as estimated by the PREDICT model, and that graphic-based questions would enhance women’s ability to accurately estimate their benefit. In preparation to formally test this hypothesis in a larger future study, we performed this pilot study to create and test questions to enhance accurate measurement of women’s perceived OS benefit from adjuvant endocrine therapy since these do not currently exist. This pilot also provided preliminary data on participants’ perceived survival benefit from adjuvant endocrine therapy.

## Methods

A two-phase exploratory sequential study design was employed with patient surveys followed by semi-structured cognitive interviews with select participants from the sample to include sufficient minority, rural and lower socioeconomic status patients [24]. Similar research designs have been commonly used in oncology research to evaluate side effect burden, quality of life (QOL), and patient reported outcomes (PRO) [25–31]. This design was chosen to gather baseline data and to elicit concrete suggestions for the optimal question(s) that would accurately measure participants’ perceived survival benefit from adjuvant endocrine therapy which may not have been captured with quantitative surveys alone.

This study enrolled English and Spanish speaking patients at the University of New Mexico Comprehensive Cancer Center from August of 2022 to March of 2023. The University of New Mexico (UNM) Health Sciences Center’s Human Research Protections Office [UNM HRPO] (#19-562) approved the study. The study enrolled women with a history of stage I-III hormone receptor positive breast cancer who were eligible for any adjuvant endocrine therapy; Tamoxifen, Anastrazole, Letrozole or Exemestane. The women must have initiated, declined, were not recommended, or discontinued the therapy within the last 5 years and be 18 years or older. Patients with Ductal Carcinoma in Situ, men, and women on adjuvant endocrine therapy beyond 5 years were excluded.

### Surveys.

Surveys were developed to collect self-reported demographics including race, ethnicity, urban vs rural status, income, education level, age, adjuvant endocrine therapy medication, and the last time the patient discussed adjuvant endocrine therapy with their provider. Additional survey components included the PROMIS Self-Efficacy for Managing Medications and Treatments questionnaire short form [32, 33] to evaluate patient confidence in choosing, managing and gathering information about medications along with a brief validated questionnaires to assess health literacy by Chew et al [34, 35]. Surveys were distributed by research coordinators to patients at the time of a regularly scheduled oncology follow-up visit and included informed consent, a study explanation, and the opportunity to decline. The surveys were completed via preloaded tablets using the REDCap platform or paper forms depending on patient preference. Spanish language versions of the survey were available in both formats and were translated by a certified Spanish translator.

### Data extraction.

Data extraction done by research coordinators from the Electronic Medical Records (EMR) included age, past medical history, tumor characteristics, type of adjuvant endocrine therapy, discontinuation reason (if applicable), menopausal status, and PREDICT model results based on that individual patient’s clinical situation.

### Interviews.

**Figure 1:**
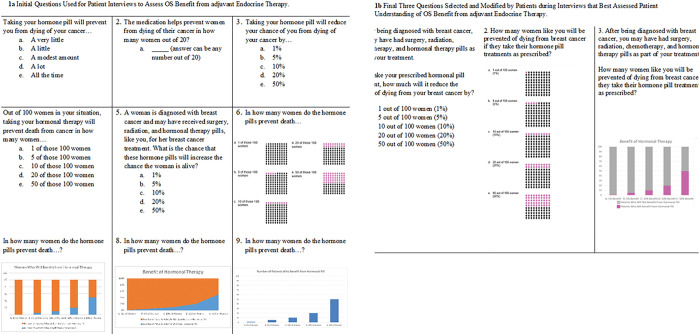
Initial and Final Questions to Measure OS Benefit from adjuvant Endocrine Therapy.

**Figure 2:**
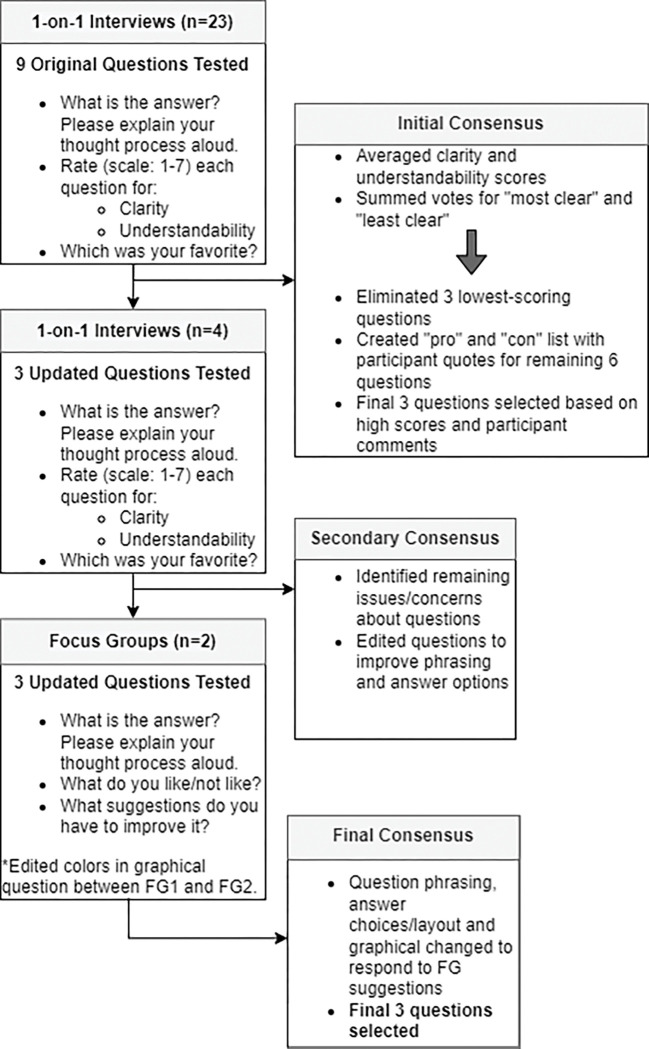
Patient Interview and Consensus Process

At the conclusion of the survey, patients could volunteer to be contacted for a phone or in-person interview. Interviewees were chosen using purposive sampling to represent a mix of rural/urban, Hispanic/Non-Hispanic, and different income levels. The interviews were completed by a Spanish and English bilingual member of the research team who had experience working with the diverse New Mexican population. The goal of the interviews was to refine and finalize survey questions designed to assess patient understanding of the benefits of adjuvant endocrine therapy. The nine questions we tested were drawn from multiple styles based on the PREDICT outputs and the COMET study [36] and included prose, fill in the blank, numerical, non-numerical, percentages, iconographs, and bar graphs (Fig. 1a). The research team developed semi-structured interview guides based on domains of interest, such as clarity and understandability, adapted from the literature [37]. The cognitive piloting occurred in three iterative rounds (Fig. 2) followed by consensus discussion meetings amongst the study team to determine next steps.

All interviews were conducted via Zoom with the camera on or in person. This allowed the interviewer to identify and respond to visual cues in body language. The interviewer asked two questions at the start of each interview, to gauge general understanding of the participant’s knowledge and priorities on the difference between cancer recurrence and dying from cancer. The interviewer then showed the test questions one by one. After reading each question to the participant, the interviewer first asked the participant to select the best answer to the question and talk through their thought process aloud. The interviewer then asked the participants to rate the question on two Likert scales from 1–7 on clarity and understanding. For example: “How clear was this question for you?” (1 = *not clear at all*, 7 = *totally clear*). Opinions were gathered through probing questions on preference of question and ways to improve test questions for clarity and understanding. The interviewer was experienced in conducting cognitive interviews and did follow-up any verbal or non-verbal reactions including hesitations, spontaneous comments, or expressions such as confusion or uncertainty per published literature [37]. Interviews lasted an average of 45 minutes. Participants received a $25 gift card for their time. We used feedback from all 27 interviews to select and make edits to the questions.

### Focus Groups:

We then conducted two focus groups (FG1 and FG2) to assess which question(s) would be the clearest and elicit the most accurate answers. We recruited participants who had already completed a cognitive piloting interview to participate in the focus groups. Focus groups were conducted only in English and via Zoom; they lasted 90 minutes each and participants were compensated with a $25 gift card for their time. Three test questions selected and modified from the interviews were the focus of each FG. Two facilitators moderated each discussion and it was explained to the participants that the original nine test questions had been modified or eliminated and why. We explained that future changes to the final three questions would be based off participants’ responses in both FGs. Approximately 30 minutes were spent discussing each test question. The final few minutes were spent gathering answers to the same overarching questions as in the interviews: “Would anything change if we asked about recurrence instead of dying and what if we asked about increasing chance of survival instead?” Changes suggested by FG1 were discussed during FG2.

## Consensus

As demonstrated in Fig. 2, consensus was an iterative process. Following each round of participant interaction, the study team met to discuss results and participant comments. The *initial consensus* process allowed us to eliminate six questions. The remaining three questions were refined during the *secondary consensus* process. During the *final consensus* following all data collection, the study team made final phrasing and layout changes to the three questions (Fig. 1b). The decision was made to include three questions instead of just one to allow for reproducibility of answers and because many patients seemed to prefer one question type over another but no one question seemed best suited for all participants.

### Endpoints.

The primary endpoint of this pilot study was to create questions to accurately and clearly measure women’s perceived OS benefit from adjuvant endocrine therapy to be used in a future study. The secondary endpoint is to measure whether or not women who initiated, declined, were not recommended, or discontinued the therapy are accurate in predicting their OS benefit compared to PREDICT modeling. Other secondary endpoints were to examine health care literacy, understanding of ability to manage medications and relationship of time interval between last discussions of adjuvant endocrine therapy benefit with accuracy of patients’ predictions.

### Data analysis.

The results of the cognitive piloting activities were primarily descriptive in nature. The input provided about each of the various question types from the individuals who engaged in interviews was summarized in tabular form, and counts and percentages were calculated. The percent of participants who provided a positive endorsement of each candidate question type was estimated. We compared a ranking of these positive endorsement proportions across question type. The data from the study surveys and the data obtained from the chart review, were descriptively summarized (means, standard deviations, etc., for quantitatively scaled data, and counts and percentages for categorical data). This included categorizations of the patients’ responses about the likely benefit from adjuvant endocrine therapy. Participants’ ratings of each question’s clarity and understanding were compared, after transformation, using linear mixed effects regression to test for differences in ratings across questions, and to identify those questions with the highest clarity and understanding, while accounting for within-person correlations. Additionally, we combined the ratings of which questions were selected to be the most or least clear into a single perceived clarity score that ranged from positive 100% (equivalent to all respondents selecting the question as being most clear) to negative 100% (equivalent to all respondents selecting the question as being the least clear), and compared these scores across the nine questions using linear mixed effects models. The difference between the patient-assessed likely benefit of treatment with adjuvant endocrine therapy was compared to the PREDICT-estimated likely benefit was assessed using a paired t-test. A further assessment of the degree to which breast cancer patients correctly identify the likely benefit of treatment with adjuvant endocrine therapy was obtained by estimating Lin’s concordance correlation coefficient [38]. This correlation coefficient is equal to a value of 1.0 when two ratings result in equal values, and is equal to zero when there is no concordance between the two ratings.

## Results

### Surveys.

**Figure 3:**
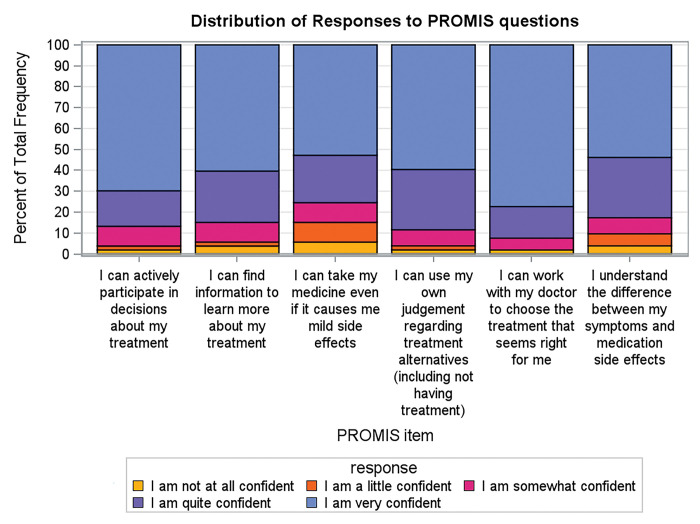
Results of PROMIS

A total of 53 patients completed the initial survey of which 42% were Hispanic, 30% rural, and 47% received $39,999 or less in household income per year (Table 1). Patients answered 88.6% of the questions asked. Patients reported average confidence about treatment and medication decisions with 70% very confident they can actively participate in treatment decisions, 58% very confident they can use their own judgement regarding treatment alternatives or not having treatment with mean score of 49.4 (95% CI 24.4–59.5) compared to a mean of 50 (standard deviation of 10) for United States general population (Fig. 3). Additionally, 61.5% of patients reported adequate health care literacy (Supplemental Table 1).

### Interviews.

Of the 53 patients who completed the initial surveys, 40 (75.5%) indicated willingness to participate in the interview, and 27 (67.5%) interviews were completed. The 13 potential participants who were not interviewed were not reachable or had scheduling conflicts. Demographics were similar for those interviewed with 44% Hispanic, 33% rural, and 47% with $39,999 or less in household income per year (Table 1). The 27 patients ranked all questions from 1–7 in terms of clarity and understanding. Linear mixed effects regression suggested that the respondents rated the nine questions differently according to their clarity and understandability (p < 0.001). Question #3 was a prose-based question and was the highest rated question with 6.8 (SD = 0.5) for clarity and 6.6 (SD = 0.8) for understanding. The next highest ranked question was #6, which used pictograms and was rated 6.6 (SD = 0.6) for clarity, and 6.6 (SD = 0.5) for understanding. Question #6 (n = 14) and Question #7 (n = 12) were the most frequently chosen as most clear. Question #7 had a significantly higher clarity score of 37% than those of questions #3, #8, #1, and #2, which had clarity scores of 0%, −11.1%, −14.8%, and − 18.5% respectively. The two questions most frequently chosen as least clear were Question #1 (n = 9) and Question #8 (n = 8). We found that there were significant differences in perceived clarity among the nine questions (p = 0.02). Each of these adjusted mean scores shared the same pooled standard error estimate of 12%. While assessing the questions during these interviews, three larger categories of problems arose: problems with the question wording, problems with the answer choices, and problems with the visual (Table 2).

**Figure 4:**
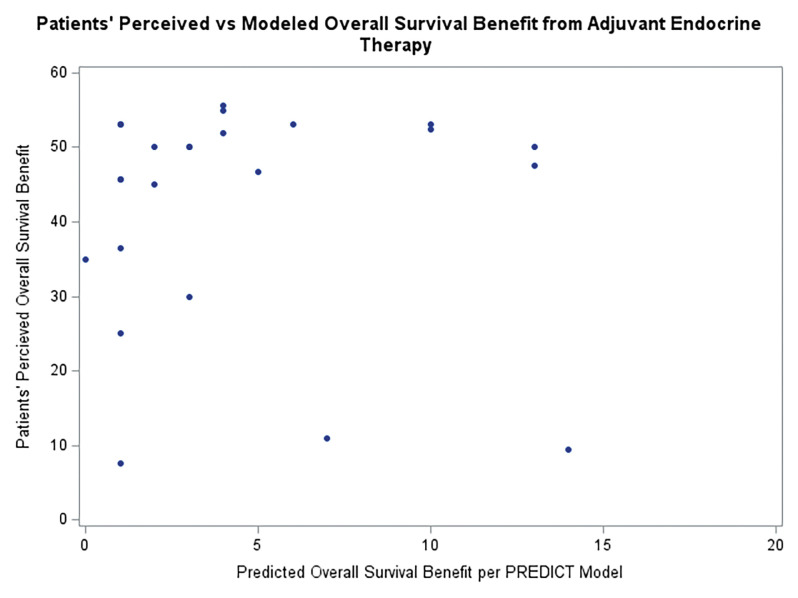
Patients Perceived vs. Modeled OS Benefit from Adjuvant Endocrine Therapy.

Each of the questions assessed in this pilot study attempted to prompt participants to estimate the perceived benefit that adjuvant endocrine therapy might provide to them. We compared the resulting self-ratings to the values calculated using the PREDICT model. Across the various question types, the patients estimated a significantly higher average benefit from adjuvant endocrine therapy on survival, at an average level of 42% (SD = 14.6) when compared to the PREDICT model which showed an average benefit of 4.4% (SD = 4.3) (Fig. 4, p < 0.001). Across the question types, the mean patient answer was consistent except for question 1, which was non-numerical prose. For questions 2–9, patients consistently estimated higher survival benefit than the PREDICT model (p < 0.001 for all). The most common answers for Question 1 was that adjuvant endocrine therapy prevent people from dying “A modest amount” (22%) and “A lot” (22%). There was no relationship detected between the question answers and when the patient last discussed adjuvant endocrine therapy with their oncologist when comparing 0–6 months vs more than 6 months ago (p = 0.22). Comparing the survey averages of the expected treatment benefit to those calculated using the PREDICT tool resulted in an estimate of Lin’s concordance correlation coefficient of −0.003, indicating essentially no agreement between the breast cancer patients’ ratings and those calculated using PREDICT.

All participants understood the difference between the cancer recurring and dying from cancer. Many participants stated that they thought more about recurrence, because they would not die from cancer unless it recurred first. We also asked participants if they would interpret the questions differently if we asked about recurrence instead of dying. This question was also often followed up by, “what if we asked about increasing survival instead?” Some participants preferred being asked in terms of dying because it was more straight-forward and “didn’t beat around the bush”. Other participants preferred the survival wording because “you see the word death a lot” and because “it gives you hope”.

Patient suggestions for how to improve patients’ understanding of the benefits of adjuvant endocrine therapy included giving participants a brochure or visual aid when they leave, teaching participants more about general medical knowledge, not being too technical, and asking medical team members to be self-aware of the difference between their own risk tolerance and their patient’s risk tolerance when recommending treatment.

## Discussion

The mixed methods study design, featuring patient surveys followed by in-depth qualitative interviews, provided nuanced understandings and preferences of these patients in underrepresented ethnic minority groups, rural and low-income patient populations. The interviews revealed that different patients perceived different questions types as being more or less clear. While most patients preferred a visual representation such as the iconograph (question #6) or bar graph (question #7), a significant minority gave that question type negative feedback and instead preferred a prose format that made the question personal by directly asking about their risk reduction (question #3). This suggests that our hypothesis that visual representation would be superior is partially correct and multiple question formats may be needed to ask patients important research questions to ensure clarity for all patients as shown in the three questions that were the final product of this study. Taken a step further, patient facing materials may ideally be represented in multiple formats to increase understanding of the risks and benefits of therapy. This has implications in both the research and clinical settings.

Patients significantly overestimated their 10-year OS benefit from adjuvant endocrine therapy compared to the estimates from the PREDICT model (42% vs 4.4%, p < 0.001). Furthermore, the patient-estimated levels of average benefit demonstrated essentially no agreement with the estimates from the PREDICT model. These findings are consistent with our hypothesis that patients do not accurately understand the OS benefit from adjuvant endocrine therapy. These patients reported adequate health care literacy along with average confidence in their medication management and treatment decisions. This adequate confidence regarding their health care literacy and average confidence in medication management is contradicted by the inaccuracy of the patients’ estimates of their OS benefit. This phenomenon of patients with confidence in their inaccurate predictions is not well described in the literature and is important for clinical care and a topic for further research. A more general trend of people being overconfident in their decisions is a well-known phenomenon which is likely related [39, 40].

Patients often do not fully understand the associated benefits and side effects of their medications [41, 42]. They often do not fully grasp the risks and benefits of medical interventions which has been well documented in research surrounding consent for surgery and clinical research [43]. Improving questionnaires meant to assess patients symptoms and ask general medical questions are an active area of research that is showing promise with the addition of graphic visualization [44, 45]. Our study adds to this body of evidence showing that women with hormone receptor positive breast cancer significantly overestimate the OS benefit from adjuvant endocrine therapy. This research also echoes previous findings that visual representation of the benefits of medications are easier to understand for most patients and adds to the literature that some patients find prose more understandable. A combination of these two question types may be the best approach. This is an important area for future research as patients who fully understand the benefits and risks of their medications can make truly informed decisions. Additionally, patients who are confident taking their medications are more likely to be adherent [46, 47]. Since poor adherence is a significant problem with adjuvant endocrine therapy leading to worse outcomes, improvement in adherence could markedly improve patient outcomes. Conversely, adjuvant endocrine therapy has significant side effects that can affect quality of life [48, 49]. Patients who have a low benefit may suffer these side effects without considering discontinuation because they believe adjuvant endocrine therapy to be much more beneficial than it actually is for them. Improving how we assess patient understanding could lead to targeted patient education which could improve outcomes for patients and potentially encourage patients to be more engaged in their health care decisions.

This study focused on OS primarily because it is considered the gold standard outcome measure in oncology. However, both OS and recurrence are important to patients as illustrated in the interviews. Similarly, our study does not take into account the significant benefit from adjuvant endocrine therapy as chemoprevention related to a subsequent cancer in either breast. Patients were aware of the difference between recurrence and OS, suggesting that this can be discussed with patients in the clinic when assessing the benefits of treatment. This is important clinically as many treatments reduce recurrence risk but have not demonstrated improvement in OS and these nuanced risk/benefit conversations can be daunting for patients and providers. The qualitative data from this study would encourage clinicians to have these nuanced conversations as patients are likely to comprehend the difference between recurrence and OS.

This study has inherent limitations. Certain populations such as African American and Asian patients were not well represented in this study, which reduces the generalizability of our findings. This work represents a single academic Comprehensive Cancer Center with a small number of providers and may not be broadly applicable to other sites or practice types. While a variety of question types for assessing patient understanding of adjuvant endocrine therapy were selected for this study based on the PREDICT model and the COMET study, potentially superior question types may have been missed for inclusion. Patients may have been thinking of and providers may have discussed relative benefit and not absolute benefit which may have led to inaccurate measurements as we measured absolute benefit in this study which we tried to make clear in the questions asked. Problems with numeracy may have also led to inaccurate results but graphical representation of questions and having multiple questions asking the same concept hopefully diminished this effect. Some women who are experiencing side effects may also report a higher benefit because they are in fact reporting the benefit they would need to have to continue to endure the side effects of their adjuvant endocrine therapy as this was documented in two interviews. The patient survey data were self-reported and subjective, and therefore may reflect self-reporting biases and/or inaccurate information. The surveys used were a combination of validated surveys and questions created solely for this study which were not previously piloted, so there may be limitations in reproducibility. In addition, the sample size was relatively small so small differences may not have been detected.

In this group of breast cancer patients with substantial representation of ethnic minority, rural and low-income groups, qualitative data showed that more than one modality of question type was needed to clearly capture participants’ perceived survival benefit from adjuvant endocrine therapy including both visual and prose-based questions. Patients also significantly overestimated their 10-year OS benefit from adjuvant endocrine therapy compared to the PREDICT model. Using this study’s results, these selected and modified questions will be used in a larger study of this patient population to confirm the above findings, examine differences among subgroups, assess providers’ estimate of benefit and providers’ estimate of patient accuracy. Future research should examine educational interventions to improve patient understanding of their adjuvant endocrine therapy, as improved understanding may support a more accurate risk / benefit assessment impacting adherence, informed treatment discontinuation and de-escalation strategies.

## Figures and Tables

**Table 1:** Patient Demographics

Demographics	All		Participated in Interview	
	N	%	N	%
	53	100%	27	50.9%
**Race and Ethnicity**				
**American Indian or Alaska Native**	3	5.7%	2	7.4%
**Asian**	2	3.8%		
**Black or African American**	1	1.9%		
**Hispanic/Latino**	22	41.5%	12	44.4%
**White**	24	45.3%	13	48.2%
**Geographic status**				
**Rural**	16	30.2%	9	33.3%
**Urban**	28	52.8%	15	55.6%
**Unsure/Missing**	9	17.0%	3	11.1%
**Annual household income**				
**Less than $10,000–39,999**	25	47.2%	14	51.9%
**$40,000 – $89,999**	16	30.2%	5	18.5%
**$90,000 +**	9	17.0%	6	22.2%
**Prefer not to answer/Missing**	3	5.7%	2	7.4%
**Highest level of education**				
**Grades 1–8**	9	17.0%	4	14.8%
**Some High School**	1	1.9%		
**High School**	11	20.8%	7	25.9%
**Bachelor’s Degree**	14	26.4%	8	29.6%
**Master’s Degree**	8	15.1%	5	18.5%
**Ph. D or Higher**	3	5.7%	1	3.7%
**Trade School**	5	9.4%	2	7.4%
**Prefer not to say/Missing**	2	3.8%		
**Sex**				
**Female**	53	100%	27	100%
**Age**				
**30–39 years**	4	7.6%	1	3.7%
**40–49 years**	6	11.3%	5	18.5%
**50–59 years**	14	26.4%	6	22.2%
**60–69 years**	13	24.5%	5	18.5%
**70–74 years**	14	26.4%	10	37.0%
**80–84 years**	2	3.8%		
**Preferred language to receive healthcare information**				
**English**	40	75.5%	19	70.4%
**Spanish**	13	24.5%	8	29.6%
**Insurance Type**				
**No Insurance/Indignant Care**	12	22.6%	7	25.9%
**Medicaid**	4	7.6%	1	3.7%
**Medicare (with any supplements)**	18	34.0%	9	33.3%
**Commercial Insurance**	16	30.2%	8	29.6%
**Veterans Affairs**	1	1.9%		
**Unsure/Don’t remember/Missing**	2	3.8%	2	7.4%
**Hormonal Therapy**				
**Received Hormonal Therapy**	44	83.0%	21	77.8%
**Tried Hormonal Therapy but Discontinued**	4	7.6%	3	11.1%
**Declined Hormonal Therapy**	5	9.4%	3	11.1%

Figure shows distribution of patient answers to questions regarding general medication understanding and management PROMIS Self-Efficacy for Managing Medications and Treatments questionnaire short form.

**Table 2:** Problems with Questions and Changes Made to Questions Based on Interviews.

Feedback on…	Sample Comments from Participants	Changes Made
Wording of the Question	• Question 5: “increase that the chance the woman is alive” it doesn’t seem like a phrase I would hear, it’s less selfexplanatory” • Question 5: “The last part of the question that I had to read over again - the question itself was a little clunky and I had to read it twice.”	We eliminated the last part of the question, and kept the context from the first part of the question.
	• Numerous Questions: Many participants found the questions more clear when we added ‘you’ or ‘your’ to the question.	Questions were updated with you or your when appropriate.
	• Numerous Questions: “Maybe when you say hormone treatment, instead say hormone pill treatment - instead of saying therapy you should say hormone pills.”	In the final questions we used the terms “hormone pill treatments” and “hormonal therapy pills.”
Answer Choices	• Question 3 and 5: “I don’t like them because of the percentage - people are not going to grasp it - I don’t like the answers.” • Question 4: “The question is hard to understand. What does 1 out of 100 mean or does it mean all the people through 100? I don’t understand.”	Added both percentages and n out of 100 to all answer choices. Combined question #3, 4, and 5 into new question.
	• Question 1: “#1 is the least clear, what I call a very little could be different for someone else.”	Eliminated question #1.
	• Question 1: “#1 was the worst because it didn’t quantify anything.”	
	• Question 2: many participants hesitated to answer, or responded with a number out of 20 followed by a percentage that didn’t match. For example, one participant said they thought it was 2 out of 20, and then later said that would be 5%.	Eliminated Question #2.
Graphic Elements	• Question 8: “I don’t think I understand the continuous graph in the context of this question. You are either benefitting or not. I don’t like this visual presentation of the answer options.”	Eliminated Question #8.
	• Question 7 and 8: Many participants did not like the color of the orange and blue. Some participants noted that the orange really highlighted how many women won’t benefit from treatment.	Changed the colors to pink to represent patients who benefit, and grey shading for the patients who don’t.
	• Question 9: Even though they are both bar graphs, participants preferred question #7 to #9 because it showed contrast by displaying both the patients who benefit from hormonal therapy along with the patients who do not benefit from hormonal therapy.	Eliminated Question #9.

Patients perceived overall survival benefit on the Y axis linked with the same patients predicted overall survival benefit per the PREDICT model on the X axis.

## Data Availability

The datasets generated during and/or analyzed during the current study are not publicly available due to fact that they could be linked to individual patients but are available from the corresponding author on reasonable request.

## References

[1] Basic Information About Breast Cancer (2023) | CDC n.d. https://www.cdc.gov/cancer/breast/basic_info/index.htm

[2] Waks AG, Winer EP (2019) Breast Cancer Treatment: A Review. JAMA 321:288–300. 10.1001/jama.2018.19323 30667505

[3] Burstein HJ, Griggs JJ, Prestrud AA, Temin S (2010) American Society of Clinical Oncology Clinical Practice Guideline Update on Adjuvant Endocrine Therapy for Women With Hormone Receptor–Positive Breast Cancer. JOP 6:243–246. 10.1200/JOP.000082 21197188 PMC2936467

[4] Pan H, Gray R, Braybrooke J, Davies C, Taylor C, McGale P (2017) 20-Year Risks of Breast-Cancer Recurrence after Stopping Endocrine Therapy at 5 Years. N Engl J Med 377:1836–1846. 10.1056/NEJMoa1701830 29117498 PMC5734609

[5] Chlebowski RT, Kim J, Haque R (2014) Adherence to Endocrine Therapy in Breast Cancer Adjuvant and Prevention Settings. Cancer Prev Res 7:378–387. 10.1158/1940-6207.CAPR-13-0389 PMC11649036 24441675

[6] Murphy CC, Bartholomew LK, Carpentier MY, Bluethmann SM, Vernon SW (2012) Adherence to adjuvant hormonal therapy among breast cancer survivors in clinical practice: a systematic review. Breast Cancer Res Treat 134:459–478. 10.1007/s10549-012-2114-5 22689091 PMC3607286

[7] Roberts MC, Wheeler SB, Reeder-Hayes K (2015) Racial/Ethnic and Socioeconomic Disparities in Endocrine Therapy Adherence in Breast Cancer: A Systematic Review. Am J Public Health 105:e4–15. 10.2105/AJPH.2014.302490 PMC4455526 25905855

[8] Mainous AG, King DE, Garr DR, Pearson WS, Race (2004) Rural Residence, and Control of Diabetes and Hypertension. Ann Fam Med 2:563–568. 10.1370/afm.119 15576542 PMC1466748

[9] Hershman DL, Shao T, Kushi LH, Buono D, Tsai WY, Fehrenbacher L (2011) Early discontinuation and non-adherence to adjuvant hormonal therapy are associated with increased mortality in women with breast cancer. Breast Cancer Res Treat 126:529–537. 10.1007/s10549-010-1132-4 20803066 PMC3462663

[10] Barron TI, Cahir C, Sharp L, Bennett K (2013) A nested case–control study of adjuvant hormonal therapy persistence and compliance, and early breast cancer recurrence in women with stage I–III breast cancer. Br J Cancer 109:1513–1521. 10.1038/bjc.2013.518 24002590 PMC3777010

[11] Berkowitz MJ, Thompson CK, Zibecchi LT, Lee MK, Streja E, Berkowitz JS (2021) How patients experience endocrine therapy for breast cancer: an online survey of side effects, adherence, and medical team support. J Cancer Surviv 15:29–39. 10.1007/s11764-020-00908-5 32804353 PMC7430212

[12] Gho SA, Steele JR, Jones SC, Munro BJ (2013) Self-reported side effects of breast cancer treatment: a cross-sectional study of incidence, associations, and the influence of exercise. Cancer Causes Control 24:517–528. 10.1007/s10552-012-0142-4 23296457

[13] Predict, Breast (2020) n.d. https://breast.predict.nhs.uk/index.html

[14] Almalki H, Absi A, Alghamdi A, Alsalmi M, Khan M (2020) Analysis of Patient-Physician Concordance in the Understanding of Chemotherapy Treatment Plans Among Patients With Cancer. JAMA Netw Open 3:e200341. 10.1001/jamanetworkopen.2020.0341 32125427 PMC7054829

[15] Hurtado-de-Mendoza A, Jensen RE, Jennings Y, Sheppard VB (2018) Understanding Breast Cancer Survivors’ Beliefs and Concerns About Adjuvant Hormonal Therapy: Promoting Adherence. J Canc Educ 33:436–439. 10.1007/s13187-017-1180-0 PMC5557694 28205022

[16] Shin DW, Cho J, Kim SY, Yang HK, Park K, Kweon S-S (2018) Patients’ and family caregivers’ understanding of the cancer stage, treatment goal, and chance of cure: A study with patient-caregiver-physician triad. Psychooncology 27:106–113. 10.1002/pon.4467 28603876

[17] Niranjan SJ, Wallace A, Williams BR, Turkman Y, Williams CP, Bhatia S (2020) Trust but Verify: Exploring the Role of Treatment-Related Information and Patient-Physician Trust in Shared Decision Making Among Patients with Metastatic Breast Cancer. J Cancer Educ 35:885–892. 10.1007/s13187-019-01538-x 31062280

[18] Risk perceptions and health behavior - PMC n (2023) d. https://www.ncbi.nlm.nih.gov/pmc/articles/PMC4525709/

[19] Freedman RA, Kouri EM, West DW, Keating NL (2015) Racial/ethnic disparities in knowledge about one’s breast cancer characteristics. Cancer 121:724–732. 10.1002/cncr.28977 25624186 PMC4946569

[20] Engelhardt EG, van den Broek AJ, Linn SC, Wishart GC, van de Rutgers EJTh AO (2017) Accuracy of the online prognostication tools PREDICT and Adjuvant! for early-stage breast cancer patients younger than 50 years. Eur J Cancer 78:37–44. 10.1016/j.ejca.2017.03.015 28412587

[21] Katusiime B, Corlett S, Reeve J, Krska J (2016) Measuring medicine-related experiences from the patient perspective: a systematic review. Patient Relat Outcome Meas 7:157–171. 10.2147/PROM.S102198 27785116 PMC5063133

[22] Faisal S, Blandford A, Potts HW (2013) Making sense of personal health information: Challenges for information visualization. Health Inf J 19:198–217. 10.1177/1460458212465213 23981395

[23] Hendriks M, Xanthopoulakis C, Vos P, Consoli S, Kustra J (2019) Data Visualization in Clinical Practice. In: Consoli S, Reforgiato Recupero D, Petković M (eds) Data Science for Healthcare: Methodologies and Applications. Springer International Publishing, Cham, pp 289–304. 10.1007/978-3-030-05249-2_11.

[24] Edmonds WA, Kennedy TD (2017) An applied guide to research designs: quantitative, qualitative, and mixed methods. Second edition, pp 196–200. Los Angeles: SAGE;

[25] Maze D, Walter RB, Merino DM, Bell TJ, O’Hara L, Peloquin F (2021) A mixed methods study exploring the role of perceived side effects on treatment decision-making in older adults with acute myeloid leukemia (AML). JCO 39:7016–7016. 10.1200/JCO.2021.39.15_suppl.7016

[26] Bluethmann SM, Murphy CC, Tiro JA, Mollica MA, Vernon SW, Bartholomew LK (2017) Deconstructing Decisions to Initiate, Maintain, or Discontinue Adjuvant Endocrine Therapy in Breast Cancer Survivors: A Mixed-Methods Study. Oncol Nurs Forum 44:E101–E110. 10.1188/17.ONF.E101-E110 28635973 PMC5507080

[27] Tolstrup LK, Pappot H, Bastholt L, Zwisler A-D, Dieperink KB (2020) Patient-Reported Outcomes During Immunotherapy for Metastatic Melanoma: Mixed Methods Study of Patients’ and Clinicians’ Experiences. J Med Internet Res 22:e14896. 10.2196/14896 32271150 PMC7180512

[28] Jaffe SA, Guest DD, Sussman AL, Wiggins CL, Anderson J, McDougall JA (2021) A sequential explanatory study of the employment experiences of population-based breast, colorectal, and prostate cancer survivors. Cancer Causes Control. 10.1007/s10552-021-01467-5 PMC8492490 34176063

[29] Wallen GR, Baker K, Stolar M, Miller-Davis C, Ames N, Yates J (2012) Palliative care outcomes in surgical oncology patients with advanced malignancies: a mixed methods approach. Qual Life Res 21:405–415. 10.1007/s11136-011-0065-7 22101861 PMC3481844

[30] LeBlanc TW, O’Donnell JD, Crowley-Matoka M, Rabow MW, Smith CB, White DB (2015) Perceptions of Palliative Care Among Hematologic Malignancy Specialists: A Mixed-Methods Study. JOP 11:e230–e238. 10.1200/JOP.2014.001859 25784580 PMC4893821

[31] Harley C, Takeuchi E, Taylor S, Keding A, Absolom K, Brown J (2012) A mixed methods approach to adapting health-related quality of life measures for use in routine oncology clinical practice. Qual Life Res 21:389–403. 10.1007/s11136-011-9983-7 21822736

[32] PROMIS Bank v1.0 - (2021) Self-Efficacy for Managing Medications and Treatments. Health Measures n.d. https://www.healthmeasures.net/search-view-measures?task=Search.search

[33] Search (2021) & View Measures n.d. https://www.healthmeasures.net/index.php?option=com_instruments&view=measure&id=575&Itemid=992 (accessed January 4.

[34] Chew LD, Bradley KA, Boyko EJ (2004) Brief questions to identify patients with inadequate health literacy. Fam Med 36:588–594 15343421

[35] Chew LD, Griffin JM, Partin MR, Noorbaloochi S, Grill JP, Snyder A (2008) Validation of Screening Questions for Limited Health Literacy in a Large VA Outpatient Population. J GEN INTERN MED 23:561–566. 10.1007/s11606-008-0520-5 18335281 PMC2324160

[36] The COMET (Comparison of Operative versus Monitoring and Endocrine Therapy) trial: a phase III randomised controlled clinical trial for low-risk ductal carcinoma in situ (DCIS), - (2023) PMC n.d. https://www.ncbi.nlm.nih.gov/pmc/articles/PMC6429899/10.1136/bmjopen-2018-026797 PMC6429899 30862637

[37] Willis G (2005) Cognitive Interviewing: A Tool For Improving Questionnaire Design.

[38] Lin LI (1989) A concordance correlation coefficient to evaluate reproducibility. Biometrics 45:255–268 2720055

[39] Pallier G, Wilkinson R, Danthiir V, Kleitman S, Knezevic G, Stankov L (2002) The role of individual differences in the accuracy of confidence judgments. J Gen Psychol 129:257–299. 10.1080/00221300209602099 12224810

[40] Moore DA, Healy PJ (2008) The trouble with overconfidence. Psychol Rev 115:502–517. 10.1037/0033-295X.115.2.502 18426301

[41] Berry DC, Raynor DK, Knapp P, Bersellini E (2003) Patients’ Understanding of Risk Associated with Medication Use. Drug-Safety 26:1–11. 10.2165/00002018-200326010-00001 12495359

[42] Graham S, Brookey J, Do Patients Understand? TPJ (2008); 12:67–69. 10.7812/TPP/07-144 PMC3037129 21331214

[43] Falagas ME, Korbila IP, Giannopoulou KP, Kondilis BK, Peppas G (2009) Informed consent: how much and what do patients understand? Am J Surg 198:420–435. 10.1016/j.amjsurg.2009.02.010 19716887

[44] Bryant MD, Schoenberg ED, Johnson TV, Goodman M, Owen Smith, Ashli Master VA (2009) Multimedia Version of a Standard Medical Questionnaire Improves Patient Understanding Across All Literacy Levels. J Urol 182:1120–1125. 10.1016/j.juro.2009.05.027 19625036

[45] Schlatmann FWM, van Balken MR, de Winter AF, de Jong I-J, Jansen CJM (2022) How Do Patients Understand Questions about Lower Urinary Tract Symptoms? A Qualitative Study of Problems in Completing Urological Questionnaires. Int J Environ Res Public Health 19:9650. 10.3390/ijerph19159650 35955002 PMC9368298

[46] Wu J-R, Moser DK, Lennie TA, Burkhart PV (2008) Medication Adherence in Patients Who Have Heart Failure: a Review of the Literature. Nurs Clin North Am 43:133–153. 10.1016/j.cnur.2007.10.006 18249229

[47] Náfrádi L, Nakamoto K, Schulz PJ (2017) Is patient empowerment the key to promote adherence? A systematic review of the relationship between self-efficacy, health locus of control and medication adherence. PLoS ONE 12:e0186458. 10.1371/journal.pone.0186458 29040335 PMC5645121

[48] Cella D, Fallowfield LJ (2008) Recognition and management of treatment-related side effects for breast cancer patients receiving adjuvant endocrine therapy. Breast Cancer Res Treat 107:167–180. 10.1007/s10549-007-9548-1 17876703

[49] Grimison PS, Stockler MR (2007) Quality of life and adjuvant systemic therapy for early-stage breast cancer. Expert Rev Anticancer Ther 7:1123–1134. 10.1586/14737140.7.8.1123 18028021

